# Synergistic effects of TGFβ2, WNT9a, and FGFR4 signals attenuate satellite cell differentiation during skeletal muscle development

**DOI:** 10.1111/acel.12788

**Published:** 2018-06-04

**Authors:** Weiya Zhang, Yueyuan Xu, Lu Zhang, Sheng Wang, Binxu Yin, Shuhong Zhao, Xinyun Li

**Affiliations:** ^1^ Key Laboratory of Agricultural Animal Genetics, Breeding, and Reproduction of the Ministry of Education & Key Laboratory of Swine Genetics and Breeding of Ministry of Agriculture Huazhong Agricultural University Wuhan China; ^2^ The Cooperative Innovation Center for Sustainable Pig Production Wuhan China

**Keywords:** age, differentiation, FGFR4, skeletal muscle satellite cell, TGFβ2, WNT9a

## Abstract

Satellite cells play a key role in the aging, generation, and damage repair of skeletal muscle. The molecular mechanism of satellite cells in these processes remains largely unknown. This study systematically investigated for the first time the characteristics of mouse satellite cells at ten different ages. Results indicated that the number and differentiation capacity of satellite cells decreased with age during skeletal muscle development. Transcriptome analysis revealed that 2,907 genes were differentially expressed at six time points at postnatal stage. WGCNA and GO analysis indicated that 1,739 of the 2,907 DEGs were mainly involved in skeletal muscle development processes. Moreover, the results of WGCNA and protein interaction analysis demonstrated that *Tgf*β*2*,* Wnt9a*, and *Fgfr4* were the key genes responsible for the differentiation of satellite cells. Functional analysis showed that TGFβ2 and WNT9a inhibited, whereas FGFR4 promoted the differentiation of satellite cells. Furthermore, each two of them had a regulatory relationship at the protein level. In vivo study also confirmed that TGFβ2 could regulate the regeneration of skeletal muscle, as well as the expression of WNT9a and FGFR4. Therefore, we concluded that the synergistic effects of TGFβ2, WNT9a, and FGFR4 were responsible for attenuating of the differentiation of aging satellite cells during skeletal muscle development. This study provided new insights into the molecular mechanism of satellite cell development. The target genes and signaling pathways investigated in this study would be useful for improving the muscle growth of livestock or treating muscle diseases in clinical settings.

## 1 INTRODUCTION

Skeletal muscle satellite cells are undifferentiated single nuclear myogenic progenitor cells, which were first discovered by Alexander Mauro with an electron microscope (Mauro, 1961). Dermomyotome has been confirmed to be the origin of muscle progenitor cells at the embryonic stage and satellite cells at the postnatal stage (Gros, Manceau, Thome & Marcelle, 2005).

The development of muscle stem cells can be divided into two important phases: embryonic and postnatal stages. At the embryonic stage, PAX7 is first expressed in the central dermomyotome and then colocalized with PAX3 in the myotome (Relaix, Rocancourt, Mansouri & Buckingham, 2005). The PAX3^+^/PAX7^+^ cells then become embryonic muscle progenitor cells, which enter into the myogenic process and are differentiated into myoblasts with the expression of MYF5 and MYOD (Bober et al., 1991; Rudnicki et al., 1993; Sassoon et al., 1989). At the postnatal stage, satellite cells originate from PAX3^+^/PAX7^+^ cells. In general, satellite cells gradually enter a quiescence state after birth (Chakkalakal, Jones, Basson & Brack, 2012; Charge & Rudnicki, 2004; Sato, Yamamoto & Sehara‐Fujisawa, 2014). Once damage occurs, satellite cells became activated and triggered the regeneration and reconstruction of skeletal muscles (Charge & Rudnicki, 2004; Collins et al., 2005; Meeson et al., 2004). A previous study indicated that muscle regeneration was attenuated due to the depletion of satellite cells in adult muscle (Fry et al., 2015). The molecular mechanism of this phenomenon remains largely unknown. Understanding the molecular characteristics of satellite cells during development would aid the study of muscle regeneration after damage, particularly for aging muscle.

Previous studies indicated that several signaling pathways participated in the myogenesis of satellite cells. The FGF signaling pathway could induce the myogenic differentiation of muscle progenitor cells (Marics, Padilla, Guillemot, Scaal & Marcelle, 2002). Pax3 could activate the FGF signaling by upregulating the expression of *Fgfr4* and *Sprouty1* (Lagha et al., 2008). The WNT and TGFβ signaling pathways could induce the fibrogenesis of satellite cells in dystrophic mice (Biressi, Miyabara, Gopinath, Carlig & Rando, 2014). The TNF, AKT, and MAPK signaling pathways participate in the proliferation and differentiation of satellite cells (Motohashi et al., 2013; Troy et al., 2012). However, the synergistic effects of different signaling pathways remain largely unknown.

This study mainly focused on the molecular mechanism of satellite cells at the postnatal stage. The results revealed that the number and differentiation capacity of satellite cells decreased during development. The results also indicated that the synergistic effects of TGFβ2, WNT9a, and FGFR4 signals were responsible for attenuating the differentiation of satellite cells during postnatal development. This study provided new insights into the molecular mechanism of satellite cell development during the postnatal stage. The genes and signaling pathways identified in this study would be useful targets for improving the muscle growth or clinical therapeutics of muscle diseases.

## 2 RESULTS

### 2.1 Dynamic expression patterns of marker genes of satellite cells during postnatal development

**Figure 1:**
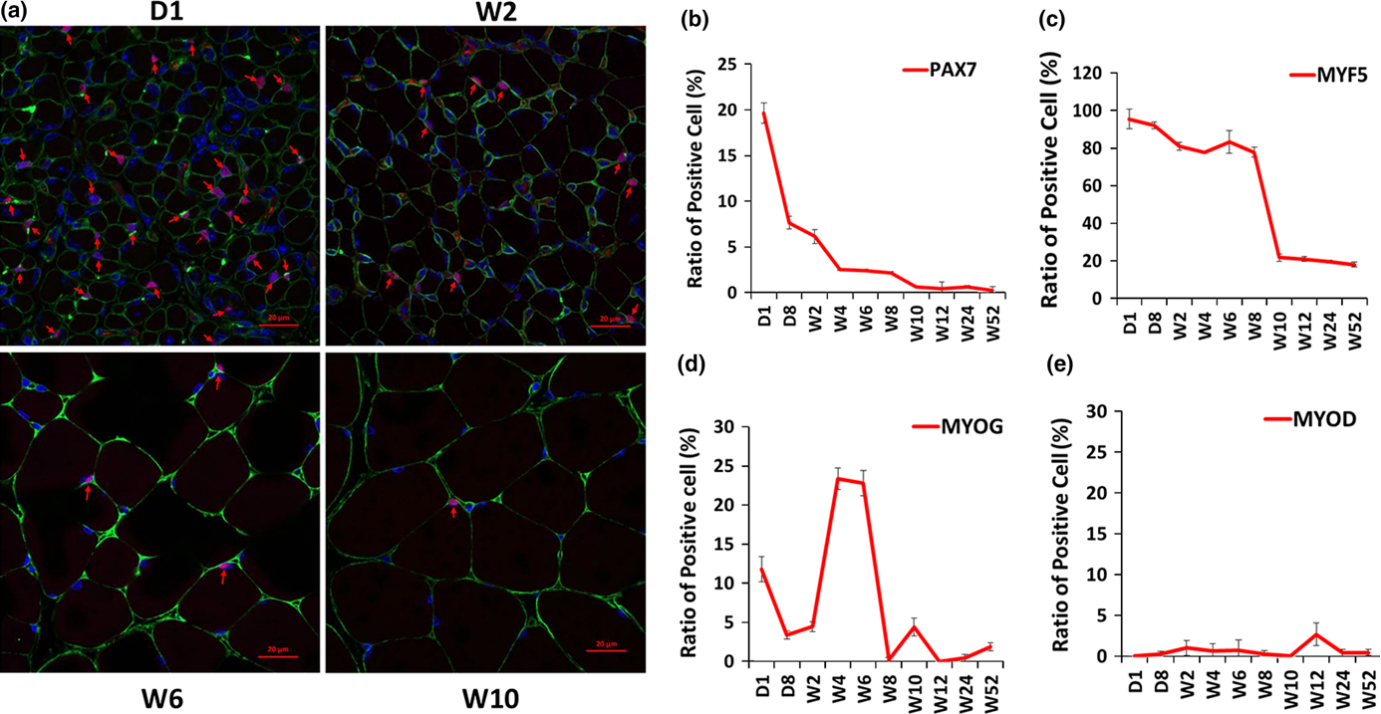
Expression patterns of myogenic factors in skeletal muscle development. Paraffin section immunofluorescence was performed to test the expression patterns of PAX7, MYF5, myogenin, and MYOD in the gastrocnemius muscle of mice at different developmental stages. (a) Confocal images of the immune stain of PAX7 (red) and laminin (green) proteins. D1, W2, W6, and W10 are shown as representatives. Nucleus was stained with DAPI (blue). PAX7‐positive cells are marked with red arrows. Scale bars: 20 μm. Magnification: 400×. (b) Change in the ratio of PAX7^+^ cells at 10 time points. (c) Change in the ratio of MYF5^+^ cells at 10 time points. (d) Change in the ratio of myogenin^+^ (MYOG) cells at 10 time points. (e) Change in the ratio of MYOD^+^ cells at 10 time points. The number of positive cells is presented as mean ± *SEM* (12 random fields are captured for each treatment group)

To investigate the development of satellite cells in postnatal skeletal muscle, we examined the expression patterns of the marker genes. The gastrocnemius muscle tissues at 10 different time points (Day 1, Day 8, Week 2, Week 4, Week 6, Week 8, Week 10, Week 12, Week 24, and Week 52) were obtained, followed by the detection of the expression of the marker genes through immunofluorescence analysis. The immunofluorescence results indicated that PAX7^+^ cells accounted for 19.7% on Day 1, and this value markedly decreased during development, accounting for <0.5% after Week 10 (Figure 1a,b, and Supporting Information Figure S1). MYF5^+^ cells only slightly decreased before Week 8 but sharply decreased at Week 10, and it remained at low levels (<20%) in the subsequent weeks (Figure 1c and Supporting Information Figure S1). Myogenin^+^ cells gradually declined from Day 1 to Week 2 but significantly increased at Week 4 and Week 6 (Figure 1d and Supporting Information Figure S1). MYOD‐positive cells maintained low levels throughout the 10 different postnatal time points (Figure 1e and Supporting Information Figure S1).

### 2.2 Differentiation capacity of satellite cells attenuated during development

**Figure 2:**
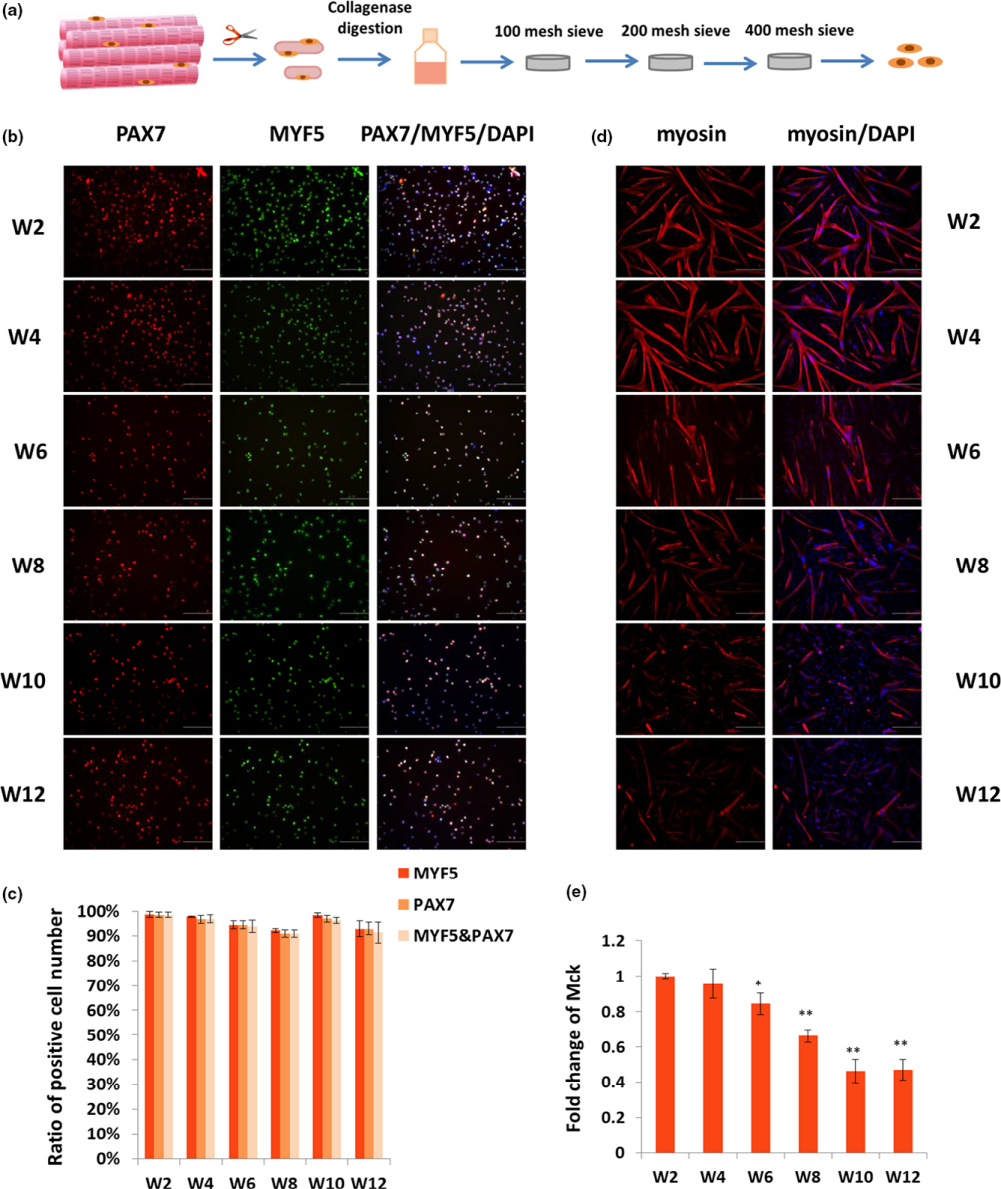
The purity and differentiation capability of isolated satellite cells. (a) Schematic of satellite cell isolation procedure. (b) Immunofluorescence staining was performed to detect the purity of separated satellite cells by staining PAX7 (red) and MYF5 (green). Cell nucleus was stained with DAPI (blue). Scale bars: 100 μm. Magnification: 200×. (c) Statistics analysis on the number of PAX7^+^/MYF5^+^ cells. The number of positive cells is presented as mean ± *SEM* (12 random fields are captured for each group). (d) Satellite cells were induced differentiation for 24 hr, and immunofluorescence staining was performed to detect myosin (red). Nucleus was stained with DAPI (blue). Scale bars: 200 μm. Magnification: 100×. (e) qPCR was performed to test the fold change in *Mck* expression in differentiated satellite cells. *Tubulin* was used as the internal control, and the relative fold change was compared to the expression in Week 2 satellite cells. Triplicate samples were analyzed for each treatment, and the results are presented as the mean ± *SEM* *p < 0.05; **p < 0.01

To further elucidate the differentiation of satellite cells, an in vitro study was performed. First, skeletal muscle satellite cells were isolated from the hindlimb muscle of mice at six different time points (Week 2, Week 4, Week 6, Week 8, Week 10, and Week 12). Immunofluorescence staining showed that more than 90% of the isolated cells were PAX7 and MYF5 double positive (Figure 2a,b). Then, the isolated satellite cells were induced with differentiation for 24 or 48 hr. The differentiation capacity was evaluated through immunofluorescence staining and quantitative polymerase chain reaction (qPCR) methods. The result of differentiation for 24 hr indicated that the myosin expression decreased with development, especially after Week 6 (Figure 2c). In the same way, the result of differentiation for 48 hr showed that the myosin expression levels were comparable at all stages, although the myotube size appeared more slender at Week 10 and Week 12 than those at the early stages (Supporting Information Figure S2A). qPCR results presented that the *Mck* expression level significantly decreased from Week 6 to Week 12 as compared to Week 2 (Figure 2d, Supporting Information Figure S2B). These results indicated that the differentiation capacity of satellite cells decreased with skeletal muscle development.

### 2.3 Transcriptome of satellite cells during skeletal muscle development

**Figure 3:**
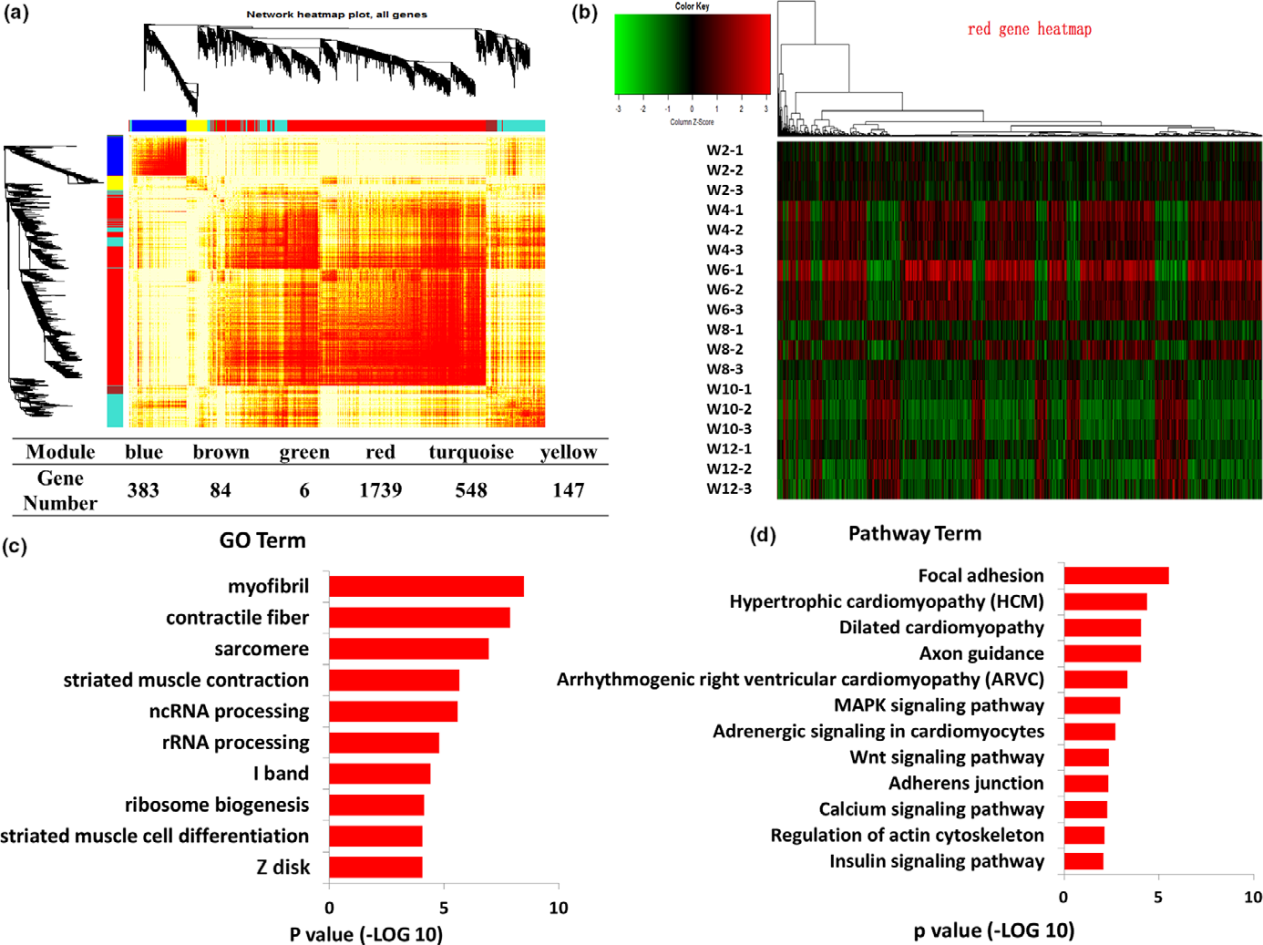
Transcriptome analysis of the satellite cells during skeletal muscle development. (a) WGCNA of the DEGs at different development stages. Branches in the hierarchical clustering dendrograms correspond to modules. Color‐coded module membership is displayed in the color bars below and to the right of the dendrograms. (b) Cluster analysis of DEGs in red module of WGCNA. (c) Significant enriched GO terms of the DEGs in red module (*p* < 0.01). (d) Significant enriched signaling pathways of the DEGs in red module (*p* < 0.01)

**Figure 4:**
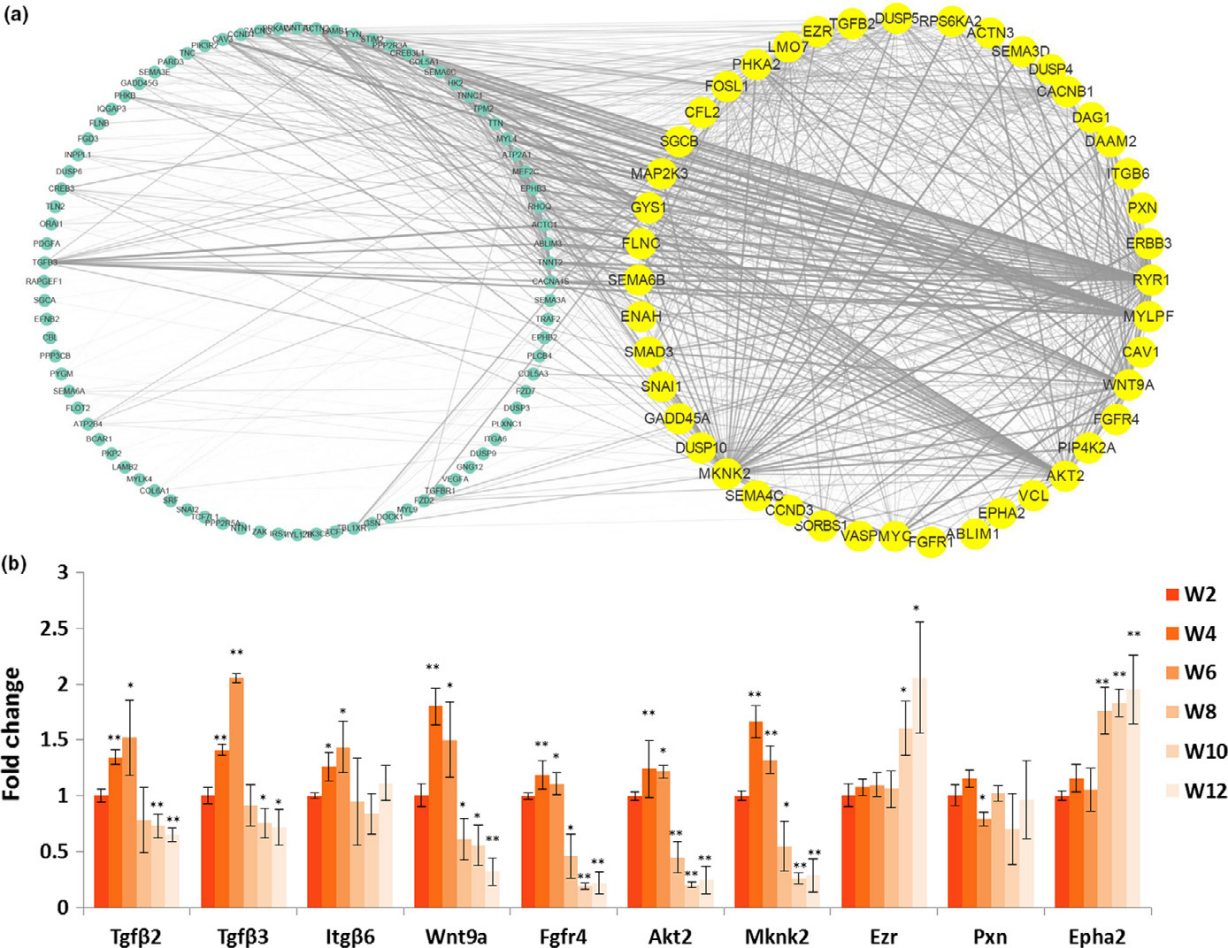
Expression correlation and qPCR detection of the DEGs in red module. (a) Network analysis of red module genes based on Weight value. Genes selected according to the number of related genes (*n* = 127) are included in the yellow circle. Other genes are shown in the circle on the left. Weight value between genes is represented by width and transparency of edges. (b) qPCR was performed to validate the expression of genes. *Tubulin* was used as the internal control, and the relative fold change was compared to the expression in Week 2 satellite cells. Triplicate samples were analyzed for each treatment, and the results are presented as the mean ± *SEM* *p < 0.05, **p < 0.01

To further understand the molecular mechanism of satellite cells during skeletal muscle development, the transcriptome profiles were detected using RNA‐seq. First, the satellite cells were isolated from the hindlimb muscle at six time points (Week 2, Week 4, Week 6, Week 8, Week 10, and Week 12). After sequencing, the differentially expressed genes (DEGs) between any two time points were further analyzed (*p* < 0.01; FDR <0.05). A total of 2,907 DEGs were identified. Furthermore, WGCNA revealed that these DEGs were enriched in six main expression modules. The largest module (Figure 3a and Supporting Information Table S3, marked in red) contained 1,739 DEGs. On the basis of the cluster analysis of the expression patterns of 1,739 genes, the six time points were regrouped into four stages, namely Week 2, Week 4 & Week 6, Week 8, and Week 10 & Week 12 (Figure 3b). GO analysis showed that the DEGs in the red module were mainly involved in skeletal muscle development processes (*p* < 0.01), including myofibril, striated muscle cell differentiation, and striated muscle contraction (Figure 3c). Pathways were analyzed using the Kyoto Encyclopedia of Genes and Genomes database, and the result indicated that the DEGs included in the red module were enriched in 12 signaling pathways (*p* < 0.01), including focal adhesion, hypertrophic cardiomyopathy, FoxO, MAPK, Wnt, and insulin signaling pathways (Figure 3d). Moreover, coexpression network of 128 genes (CPM >20) in these 12 signaling pathways were drawn based on WGCNA. The coexpression relationship of the 128 genes was represented by width and transparency of edges, and the wider and darker of edge meant the higher correlation between two genes. According to the network, 44 genes had most coexpressed genes (Figure 4a, highlighted in yellow). Moreover, the expression levels of 10 key node genes were selected for qPCR validation. The qPCR results were highly positively correlated with the RNA‐seq results (*R* > 0.8; Supporting Information Figure S3). *Tgf*β*2*,* Tgf*β*3*,* Wnt9a*,* Fgfr4*,* Akt2*, and *Mknk2* were significantly upregulated at Week 4 and Week 6; however, they were downregulated from Week 8 to Week 12 (Figure 4b). The protein interaction network of the 128 genes was drawn using String software (confidence >0.7; Supporting Information Figure S4). Therefore, the Tgfβ, Wnt, and Fgf signaling pathways were identified as the key factors responsible for the differentiation of satellite cells.

### 2.4 Tgfβ2 and Tgfβ3 genes inhibited the differentiation of satellite cells

**Figure 5:**
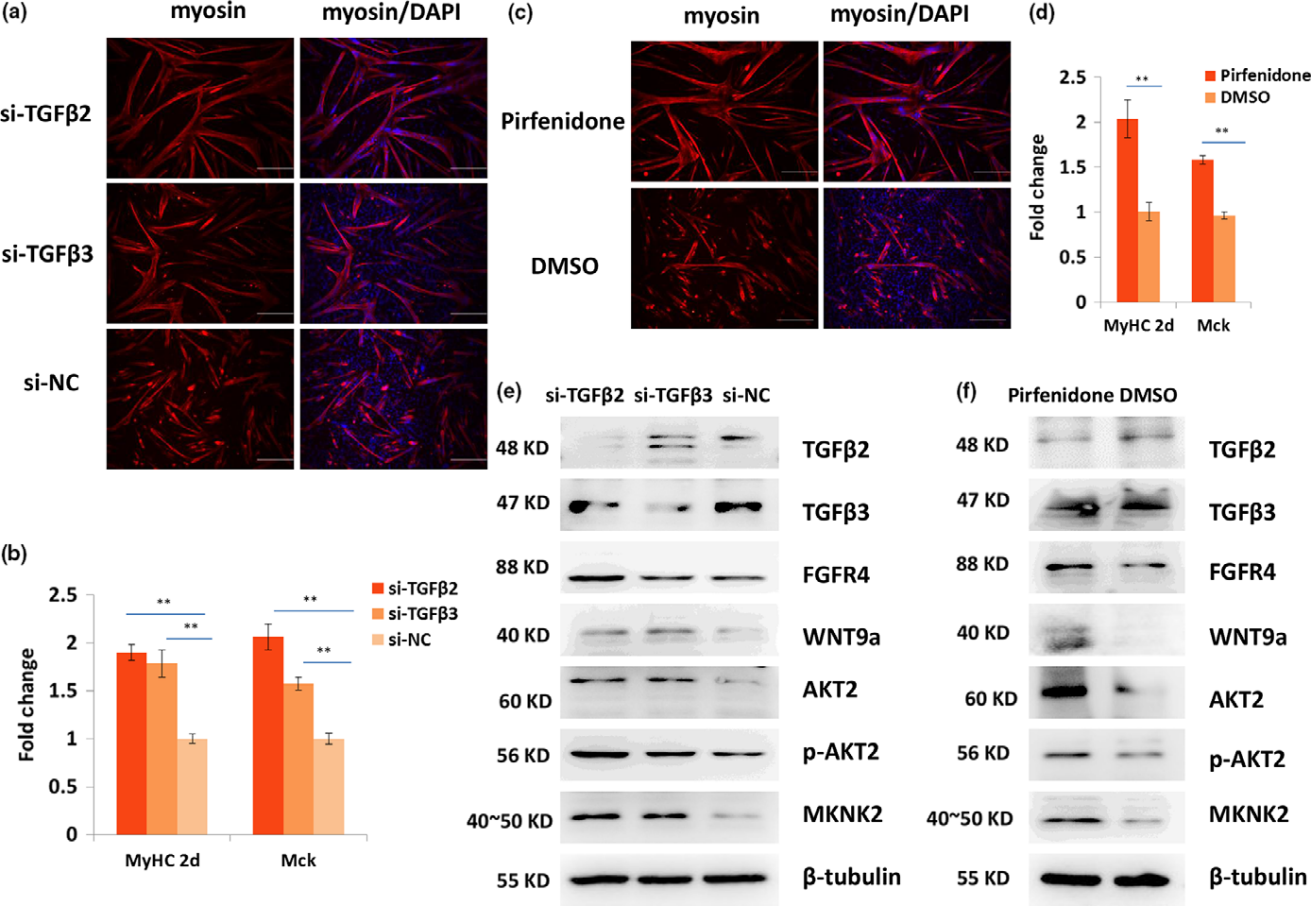
TGFβ2 and TGFβ3 negatively regulate the differentiation of satellite cells. (a) Immunofluorescence staining of myosin (red) in the 24‐hr differentiated satellite cells when Tgfβ2 or Tgfβ3 was inhibited using RNAi. Nucleus was stained with DAPI (blue) Scale bars: 200 μm. Magnification: 100×. (b) qPCR results of the expression change in *MyHc2d* and *Mck* when TGFβ2 or TGFβ3 was inhibited using siRNA. (c) Immunofluorescence staining of myosin (red) when TGFβ2 and TGFβ3 were inhibited using pirfenidone. Nucleus was stained with DAPI (blue) Scale bars: 200 μm. Magnification: 100×. (d) qPCR results of the expression change in *MyHC2d* and *Mck* when TGFβ2 and TGFβ3 were inhibited using pirfenidone. (e, f) Western blot results of TGFβ2, TGFβ3, WNT9a, FGFR4, AKT2, p‐AKT2, and MKNK2 in proliferative satellite cells when TGFβ2 and TGFβ3 was inhibited using siRNA (e) or pirfenidone (f). *Tubulin* was used as the internal control for qPCR and western blot. Triplicate samples were analyzed for each treatment, and the results are presented as the mean ± *SEM* *p < 0.05; **p < 0.01

The roles of Tgfβ2 and Tgfβ3 in the differentiation of satellite cells were further investigated. Satellite cells were first isolated from the hindlimb muscle of 4‐week‐old mice. Then, the expression levels of *Tgf*β*2*,* Tgf*β*3*,* Akt2*, and *Mknk2* were detected during proliferation and differentiation using qPCR. The results showed that *Tgf*β*2*,* Tgf*β*3*,* Akt2*, and *Mknk2* were significantly upregulated during differentiation (Supporting Information Figure S5A). Immunofluorescence staining results revealed that after differentiation for 24 hr, myosin increased in si‐Tgfβ2‐ and si‐Tgfβ3‐transfected satellite cells as compared to the negative control (NC) (Figure 5a). The qPCR results showed that the expression levels of *MyHC2d* and *Mck* genes were increased in the si‐Tgfβ2‐ and si‐Tgfβ3‐transfected groups (Figure 5b). The inhibition of TGFβ2 and TGFβ3 through their inhibitor pirfenidone exerted similar effects on the small interfering RNA (siRNA) (Figure 5c,d). In addition, the western blot results showed that FGFR4, WNT9a, MKNK2, and AKT2 were increased in proliferation satellite cells when TGFβ2 and TGFβ3 were inhibited by pirfenidone or siRNA (Figure 5e,f). Furthermore, PAX7 decreased and MYOD increased when TGFβ2 and TGFβ3 were inhibited (Supporting Information Figure S5B,C). Therefore, TGFβ2 and TGFβ3 can inhibit the differentiation of satellite cells.

### 2.5 FGFR4 and WNT9a played important roles in the differentiation of satellite cells

**Figure 6:**
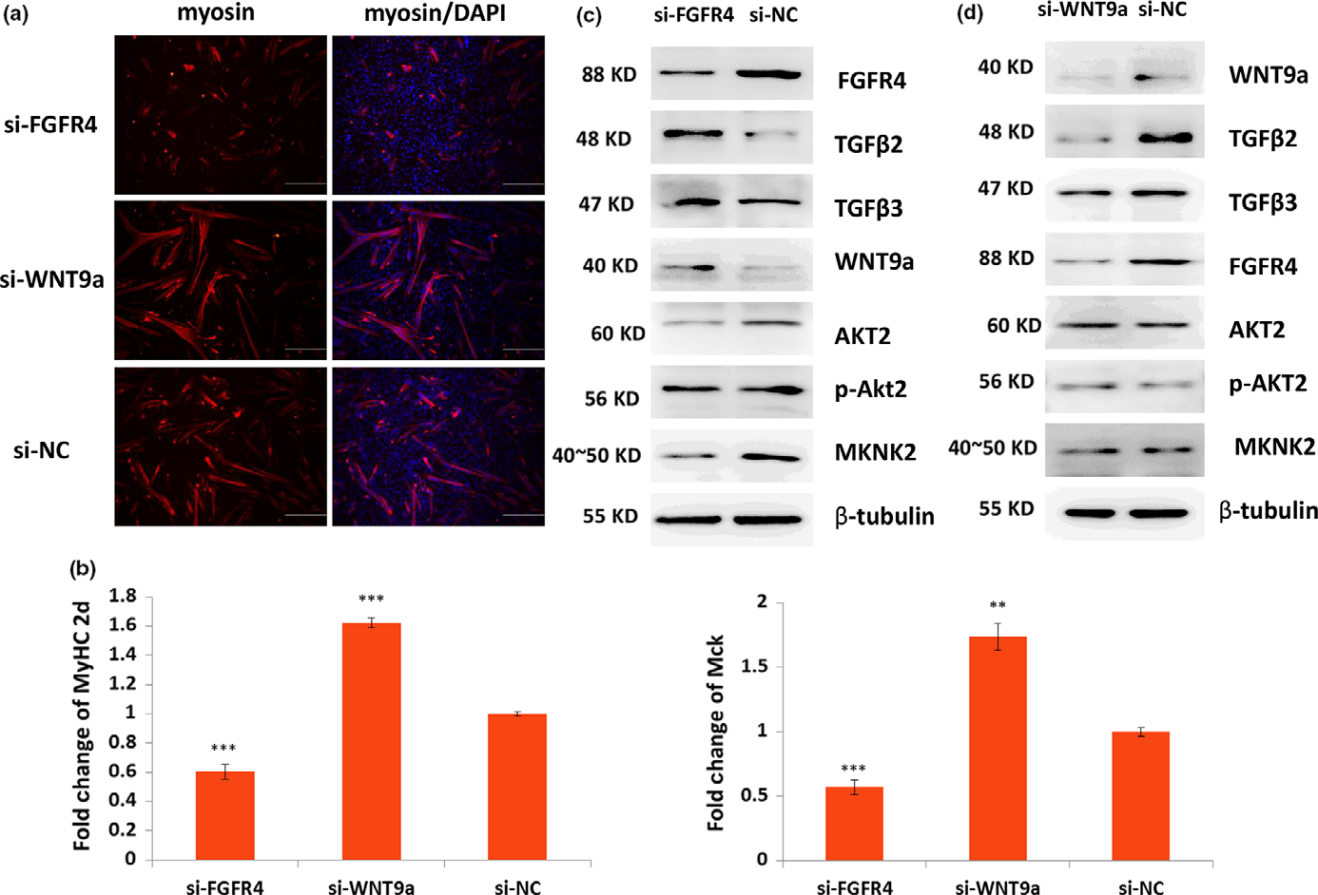
Fgfr4 and WNT9a positively and negatively regulated, respectively, the differentiation of satellite cells. (a) Immunofluorescence staining results of expression change in myosin (red) in the 24‐hr differentiated satellite cells when FGFR4 or WNT9a was inhibited using siRNA. Nucleus was stained with DAPI (blue). Scale bars: 200 μm. Magnification: 100×. (b) qPCR results of the expression change in *MyHC2d* and *Mck* when FGFR4 or WNT9a was inhibited. (c, d) Western blot results of TGFβ2, TGFβ3, WNT9a, FGFR4, AKT2, p‐AKT2, and MKNK2 in proliferative satellite cells when FGFR4 or WNT9a was inhibited. *Tubulin* was used as the internal control for qPCR and western blot. Triplicate samples were analyzed for each treatment, and the results are presented as the mean ± *SEM* *p < 0.05; **p < 0.01

The roles of FGFR4 and WNT9a in the differentiation of satellite cells were investigated. During differentiation, *Wnt9a* and *Fgfr4* genes were significantly upregulated (Supporting Information Figure S5A). Immunofluorescence results showed that myosin decreased when FGFR4 was suppressed and increased when WNT9a was suppressed (Figure 6a). qPCR results showed that *MyHC2d* and *Mck* genes were significantly decreased in the si‐Fgfr4‐transfected group but were significantly upregulated in the si‐Wnt9a‐transfected group (Figure 6b). In addition, the western blot results indicated that TGFβ2, TGFβ3, and WNT9a increased, whereas AKT2 and MKNK2 decreased, when FGFR4 was inhibited in proliferative satellite cells (Figure 6c). Moreover, TGFβ2, TGFβ3, and FGFR4 decreased, whereas AKT2 and MKNK2 remained stable when Wnt9a was inhibited (Figure 6d). PAX7 was increased, whereas MYOD was decreased, when FGFR4 was inhibited in the satellite cells (Supporting Information Figure S5D,E). These results indicated that FGFR4 and WNT9a played important roles in the differentiation of satellite cells.

### 2.6 Inhibition of TGFβ2 enhanced the differentiation of satellite cells and the regeneration of skeletal muscle in vivo

**Figure 7:**
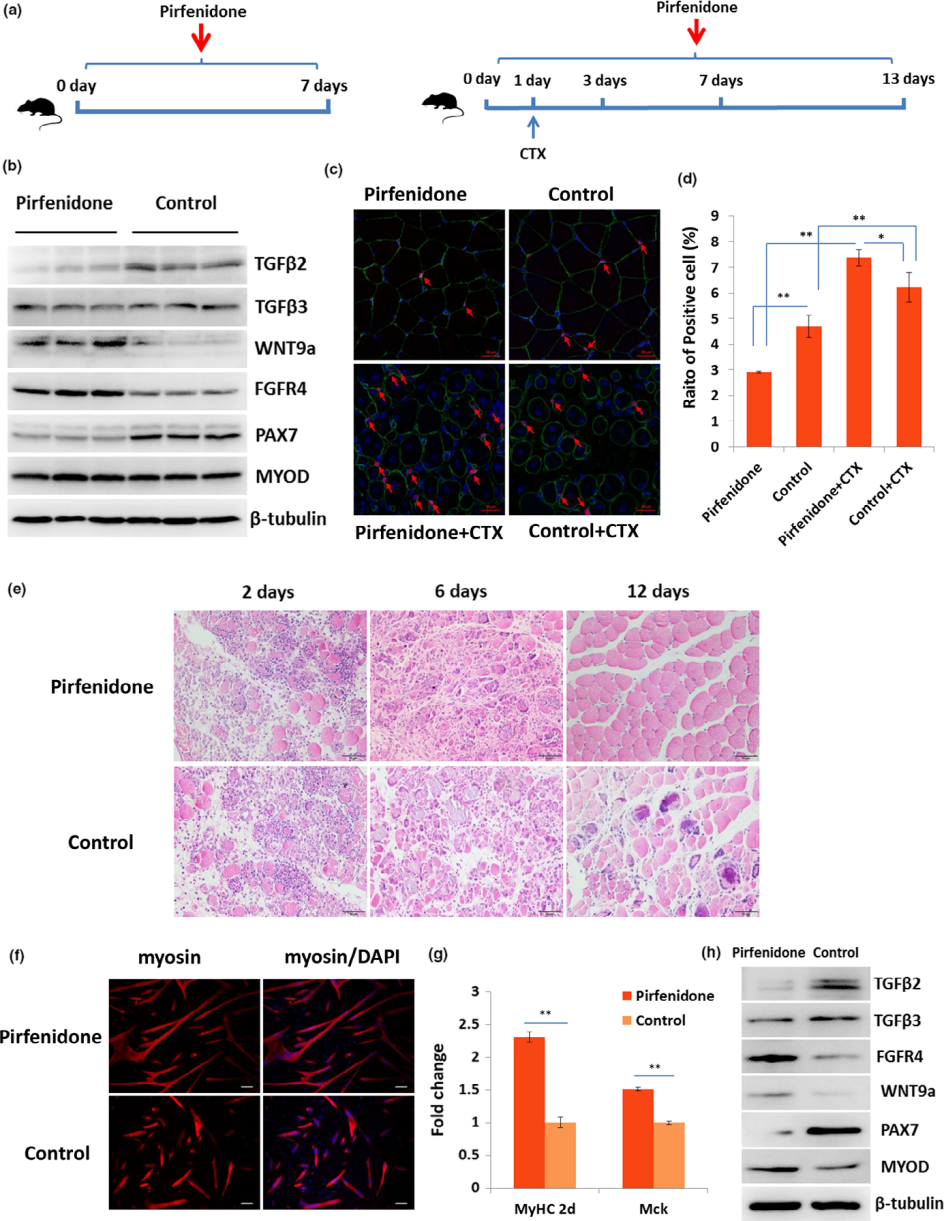
Inhibition of TGFβ2 enhanced the differentiation of satellite cells and regeneration of skeletal muscle in vivo. (a) Scheme of orally administered of pirfenidone. (b) Western blot results of TGFβ2, TGFβ3, WNT9a, FGFR4, PAX7, and MYOD in gastrocnemius muscle from pirfenidone‐treated and control mice. (c) Confocal images of the immune stain of PAX7 (red) and laminin (green) proteins in gastrocnemius muscle from pirfenidone‐treated and control mice. Nucleus was stained with DAPI (blue). PAX7‐positive cells were marked with red arrows. Scale bars: 20 μm. Magnification: 400×. (d) The statistical analysis of PAX7^+^ cell number (12 random fields are captured for each group). (e) H&E staining of the injured muscle at different time points. (f) Western blot results of TGFβ2, TGFβ3, WNT9a, FGFR4, PAX7, and MYOD in satellite cells isolated from pirfenidone‐treated and control mice. (g) Immunofluorescence staining of myosin (red) in the 24‐hr differentiated satellite cells isolated from pirfenidone‐treated and control mice. The nucleus was stained with DAPI (blue). Scale bars: 200 μm. Magnification: 100×. (h) qPCR results of the expression change in *MyHC2d* and *Mck* in the 24‐hr differentiated satellite cells. *Tubulin* was used as the internal control, and the relative fold change was compared to the control group. Triplicate samples were analyzed for each treatment, and the results are presented as the mean ± *SEM* *p < 0.05; **p < 0.01

To further verify the roles of TGFβ2 in the differentiation of satellite cells in vivo, a TGFβ2‐inhibited mice model was created via oral administration of pirfenidone daily (Figure 7a). Then, the gastrocnemius muscle was obtained from the mice model on Day 7 of pirfenidone treatment. The western blot results showed that the TGFβ2 in the gastrocnemius muscle tissue was markedly lower than that in the control mice. In addition, FGFR4, WNT9a, PAX7, and MYOD were upregulated in the gastrocnemius muscle of pirfenidone‐treated mice (Figure 7b). The pirfenidone‐treated and control groups were compared in terms of the number of Pax7^+^ cells. The immunofluorescence results indicated that Pax7^+^ cells were significantly reduced in the pirfenidone‐treated mice (*p* < 0.01). A muscle‐damaged mice model was created through the injection of cardiotoxin (CTX). On Day 6 of CTX injection, the number of Pax7^+^ cells in the damaged gastrocnemius muscle was significantly higher than that in uninjured muscle. Moreover, the number of Pax7^+^ cells in the pirfenidone‐treated mice was significantly higher than that in the control mice (Figure 7c,d). The hematoxylin and eosin (H&E) staining results showed that the skeletal muscle regeneration in the pirfenidone‐treated mice was preceded than that in the control mice on Day 6 and Day 12 after CTX injection, respectively (Figure 7e). The satellite cells were isolated on Day 7 of pirfenidone administration. The western blot results showed that under pirfenidone treatment, TGFβ2 was obviously inhibited, whereas FGFR4, WNT9a, PAX7, and MYOD were upregulated (Figure 7f). Moreover, the immunofluorescence and qPCR results revealed that myosin, *MyHC2d*, and *Mck* were upregulated in the pirfenidone treatment group relative to their levels in the control group (Figure 7g,h). These results indicated that TGFβ2 can regulate the differentiation of satellite cells and the regeneration of skeletal muscle by interacting with FGFR4 and WNT9a in mice.

## 3 DISCUSSION

Satellite cells play important roles in the development and regeneration of skeletal muscle. To reveal the development of satellite cells, we first analyzed the expression patterns of the marker genes of satellite cells during skeletal muscle development. Then, we examined the differentiation capacity of satellites cells at different ages. Our results indicated that the differentiation capacity of satellite cells was attenuated with development. Moreover, the molecular mechanism of the attenuation of differentiation was assessed by performing transcriptome and functional analyses. We concluded that the synergistic effects of TGFβ2, WNT9a, and FGFR4 signals were responsible for attenuating the differentiation of skeletal muscle satellite cells.

In the study, we found that the number of PAX7^+^ cells was rapidly decreased at infancy and then remained at very low levels at the adult stage. In addition, previous studies demonstrated that the self‐renewal ability and the number of PAX7‐positive satellite cells decreased with age (Bernet et al., 2014; Collins, Zammit, Ruiz, Morgan & Partridge, 2007). The number of myogenin^+^ cells was increased in Week 4 and Week 6. Therefore, we speculated that satellite cells were rapidly consumed after birth, leading to the accumulation of the nucleus of the myofiber at the early postnatal stage. The myofiber then entered a period of rapid growth from Week 4 to Week 6.

Transcriptome analysis revealed that the development of satellite cells can be divided into four stages. WGCNA indicated that TGFβ2, TGFβ3, WNT9a, and FGFR4 signals were important for the development of satellite cells. These signals had a coexpression relationship with many effector genes of skeletal muscle development, including *Ryr1*,* Mylpf*,* Akt2*, and *Mknk2*. MYLPF is a marker of fast skeletal muscle, and RYR1 functions as a calcium release channel in muscle contraction (Eltit et al., 2010; Wang et al., 2007). AKT2 could enhance myogenic differentiation, and MKNK2 could promote cell proliferation (Heron‐Milhavet, Mamaeva, Rochat, Lamb & Fernandez, 2008; Maimon et al., 2014; Teo et al., 2015). All these effector genes were downregulated during development, implying that the genes and signals identified through WGCNA were important for the differentiation of satellite cells.

The upstream genes in the signals should theoretically have more important functions. Thus, the functions of the four identified upstream genes, namely FGFR4, TGFβ2, TGFβ3, and WNT9a, were further investigated. FGFR4 could promote the differentiation of satellite cells, whereas TGFβ2, TGFβ3, and WNT9a displayed an opposite function. In vitro analysis showed that TGFβ2 and WNT9a could increase PAX7 and inhibit MYOD at the protein level, whereas FGFR4 could downregulate PAX7 and upregulate MYOD at the protein level in satellite cells. In vivo analysis indicated that the inhibition of TGFβ2 could downregulate the expression of PAX7 and promote the regeneration of skeletal muscles. Previous studies indicated that the activation of quiescent satellite cells was accompanied by reduced PAX7 and increased MYOD (Kostallari et al., 2015; Sato et al., 2014). The overexpression of TGFβ2 and TGFβ3 can decrease the myogenic differentiation of myoblasts (de Mello, Streit, Sabin & Gabillard, 2015; Schabort, van der Merwe & Niesler, 2011). Moreover, the knockout of FGFR4 attenuated the skeletal muscle regeneration (Zhao et al., 2006). Therefore, TGFβ2, WNT9a, and FGFR4 could regulate the skeletal muscle development by adjusting the activation status of satellite cells.

**Figure 8:**
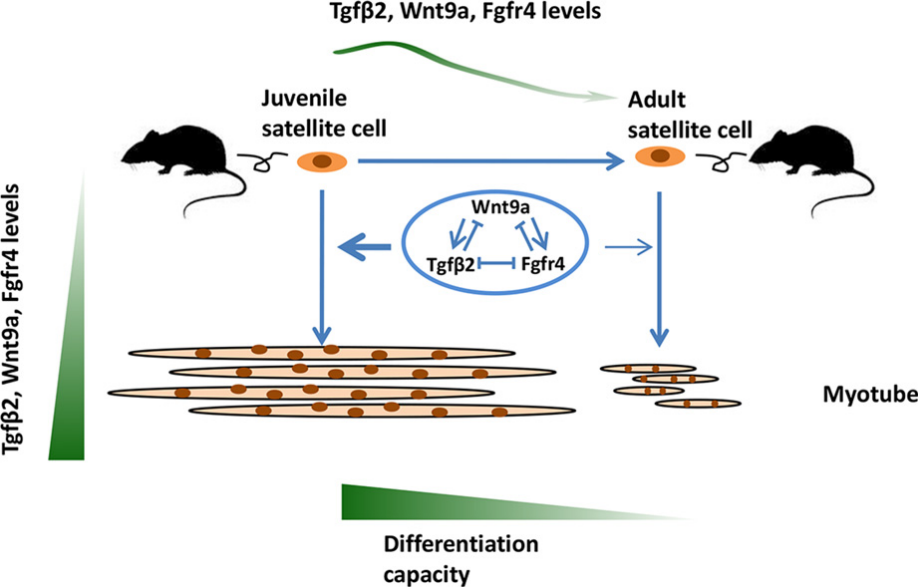
Schema graph of TGFβ2, WNT9a, and FGFR4 signals in the differentiation of satellite cells during skeletal muscle development

Furthermore, the regulation of TGFβ2, TGFβ3, WNT9a, and FGFR4 was investigated. The results indicated that FGFR4 and TGFβ2 inhibited each other in satellite cells. Both TGFβ2 and FGFR4 inhibited WNT9a, whereas WNT9a promoted the expression of TGFβ2 and FGFR4 in the satellite cells. TGFβ3 had a similar but weaker effect to TGFβ2. In vivo analysis confirmed that TGFβ2 negatively regulated FGFR4 and Wnt9a. Basing on these results, we drew a schema graph to demonstrate the synergistic effects of TGFβ2, WNT9a, and FGFR4 on the differentiation of satellite cells (Figure 8). The interaction among these three genes ultimately reached a balance. This balance remained at high levels and had a stronger effect at the early stage than at the adult stage because of the attenuation of the differentiation capacity of satellite cells during development.

## 4 MATERIALS AND METHOD

### 4.1 Mice

All C57BL/6 mice used in this study were obtained from Hubei Center for Disease Control and Prevention (Wuhan, China). All experiments were performed in accordance with the Guide for the Care and Use of Laboratory Animals (Institute of Laboratory Animal Resources, Commission on Life Sciences, National Research Council, 1996), and the protocols were approved by the Hubei Province Committee on Laboratory Animal Care (HZAUMU2013‐0005).

### 4.2 Isolation and culture of muscle satellite cells

Satellite cells were isolated from the hindlimb muscle tissues at six different time points (postnatal Week 2, Week 4, Week 6, Week 8, Week 10, and Week 12; *n* = 8~12). Satellite cell isolation method referred that described in previous study (Lu et al., 2008). The muscle tissues were digested for 60–90 min with collagenase I (2 mg/ml; Sigma, USA, C1639) at 37°C. The dissociated suspension was sifted through 100, 200, and 400 mesh sieves. Then, the suspension was washed with RPMI 1640 medium, resuspended by growth medium with 15% fetal calf serum (Gibco, USA, 10082‐147), chick embryo extract (GEMINI, USA, 100‐163p), basic fibroblast growth factor (Life, USA, 13256‐029; 0.25 μg/100 ml), and RPMI 1640 medium. The suspension was plated on a normal dish and then transferred to a dish coated with Matrigel (BD, USA, 356234) after 2.5 hr. The satellite cells were cultured at 37°C in a cell incubator with 5% CO_2_ until they converged to 60%. Then, the second differential attachment experiment was performed. The differentiation medium was composed of Dulbecco's modified Eagle's medium (DMEM) and 3% (v/v) horse serum (Gibco). Pirfenidone (Selleck, USA; 20 μg/ml; Burghardt et al., 2007) was used to stimulate the satellite cells continuously for 24 hr.

### 4.3 Cell transfection

For RNAi assay, the isolated satellite cells were transfected with siRNA using Lipofectamine 2000 (Invitrogen, USA) in accordance with the manufacturer's recommendations after the cells converged to approximately 60%. The siRNA and the scrambled negative control were provided by RIBOBIO (RIBOBIO, P.R.C).

### 4.4 Animal assay

Pirfenidone (Selleck, S2907) was dissolved by a vehicle containing 2% DMSO (Sigma, D2650) and 30% polyethylene glycol 300 (PEG 300; Sigma, 90878). Four‐week‐old mice were randomly divided into two groups (*n* = 11 for each group). Pirfenidone (250 mg kg^−1^ day^−1^; Nakazato, Oku, Yamane, Tsuruta & Suzuki, 2002) or vehicle (control) was orally administered daily for 7 days. Then, the gastrocnemius muscle was acquired for protein extraction and immunofluorescence. For satellite cell isolation, the total muscle of the hind leg was used (*n* = 8). Regeneration assay was also performed. In the same way, 4‐week‐old mice were divided into two groups (*n* = 9 for each group). Pirfenidone (250 mg kg^−1^ day^−1^) or vehicle (control) was orally administered daily. After 1 day of the first pirfenidone treatment, CTX was injected into gastrocnemius with 100μl of 10μM (Qiu et al., 2016). Then, the gastrocnemius was isolated on Day 2, Day 6, and Day 12 after CTX injection to analyze the tissue morphology.

### 4.5 Immunofluorescence of cell

The satellite cells were collected when they converged to approximately 60% or induced differentiation for 24 or 48 hr, washed twice with phosphate‐buffered saline (PBS), and fixed in 4% paraformaldehyde for 15 min. Then, the satellite cells were washed twice with PBS, incubated in an ice‐cold 0.25% Triton X‐100 at room temperature for 10 min, and again washed thrice. The cells were incubated in blocking solution (3% bovine serum albumin, 0.3% Triton X‐100, 10% fetal bovine serum in PBS) at room temperature for 2 hr and then incubated in primary antibody at 4°C overnight. The primary antibodies for immunofluorescence staining are shown in Supporting Information Table S1. The cells were washed thrice with PBS and then incubated with anti‐mouse IgG (H+L), F (ab’) 2 Fragment (Alexa Fluor^®^ 555 Conjugate; CST, USA, 4409), and anti‐rabbit IgG (H+L), F(ab’) 2 Fragment (Alexa Fluor^®^ 488 Conjugate; CST, 4412) for 2 hr. The cell nucleus was washed thrice with PBS and stained with 4′, 6‐diamidino‐2‐phenylindole (DAPI) (Wei et al., 2013). Images were captured using a Nikon Eclipse TE2000‐S system (Nikon, Japan).

### 4.6 Immunofluorescence of tissue sections

The gastrocnemius muscle was dissected form pirfenidone‐treated mice and mice of different ages (postnatal Day 1, Day 8, Week 2, Week 4, Week 6, Week 8, Week 10, Week 12, Week 24, and Week 52). After fixation with 4% paraformaldehyde, the skeletal muscle samples were embedded in paraffin, and 4‐um‐thick serial sections were obtained. After deparaffinization, citric acid buffer (PH6.0) microwave antigen retrieval method was used. Additional immunofluorescence staining was performed according to the cell immunofluorescence experiment. Images were captured using both Nikon ECLIPSE TE2000‐S system (Nikon) and confocal microscopy (ZEISS, Germany).

### 4.7 RNA‐seq

The total RNA was extracted from isolated satellite cells at six different time points using RNeasy Mini Kit (Qiagen, Germany, 74106) in accordance with the manufacturer's instructions. Qualified total RNA was further purified using the RNAClean XP Kit (Beckman Coulter, Inc., Kraemer Boulevard Brea, CA, USA, A63987) and the RNase‐Free DNase Set (Qiagen, 79254). RNA and the library preparation integrity were verified with an Agilent Bioanalyzer 2100 (Agilent Technologies, Santa Clara, CA, USA). We accomplished the cluster and first dimension sequencing primer hybridization on cBot of Illumina sequencing machine in accordance with the cBot User Guide. Sequencing was performed by Shanghai Biotechnology Corporation (P.R.C). Edger, which is an R package, was used to screen the DEGs.

### 4.8 qPCR

Reverse transcription was performed to initiate cDNA synthesis using the Prime ScriptTM RT Reagent Kit with gDNA Eraser (TAKARA BIO INC, Otsu, Shiga, Japan). THUNDERBIRD SYBR qPCR Mix (TOYOBO, Japan) was used for qPCR, and the results were monitored using a CFX384 Real‐Time PCR Detection System (Bio‐Rad, USA). All primer sequences are listed in the supplementary data (Supporting Information Table S2).

### 4.9 Western blot

The Mammalian Protein Extraction Reagent (Pierce, USA) was used to obtain the protein lysate. SDS‐PAGE was used to separate the proteins, and a Mini Trans‐Blotting Cell (Bio‐Rad) was used to transfer protein onto polyvinylidene fluoride membranes (Millipore, USA). Primary antibodies (Supporting Information Table S1) and horseradish peroxidase (HRP)‐labeled anti‐rabbit‐IgG or anti‐mouse‐IgG secondary antibodies (Beyotime, P.R.C.) were used for immunoblotting. An Image Quant LAS4000 mini (GE Healthcare Bio‐Sciences, USA) was used to detect the signal produced by the Immobilon Western Chemiluminescent HRP Substrate (Millipore).

### 4.10 Statistical analysis

All results are expressed as mean ± *SEM*. Unpaired Student's *t* tests were used to determine the statistical significance, and *p* < 0.05 indicated a significant difference.

## CONFLICT OF INTEREST

The authors declare that they have no competing or financial interests.

## AUTHOR CONTRIBUTIONS

Weiya Zhang, Lu Zhang, Yinlong Liao, Sheng Wang, and Binxu Yin conducted the experiments and prepared the materials involved in this study. Yueyuan Xu performed the bioinformatics analysis. Xinyun Li conceived this study. Shuhong Zhao, Xinyun Li, and Weiya Zhang participated in its design and coordination. Xinyun Li, Weiya Zhang, and Yueyuan Xu contributed to the analysis and interpretation of the data. Weiya Zhang drafted the manuscript. Shuhong Zhao and Xinyun Li helped to revise the manuscript. All authors read and approved the final manuscript.
