# Supplementary figures and images for: Synergistic effects of TGFβ2, WNT9a, and FGFR4 signals attenuate satellite cell differentiation during skeletal muscle development
Source: Aging Cell. 2018 Jun 4;17(4):e12788. doi: 10.1111/acel.12788 (PMC6052404; doi:10.1111/acel.12788)

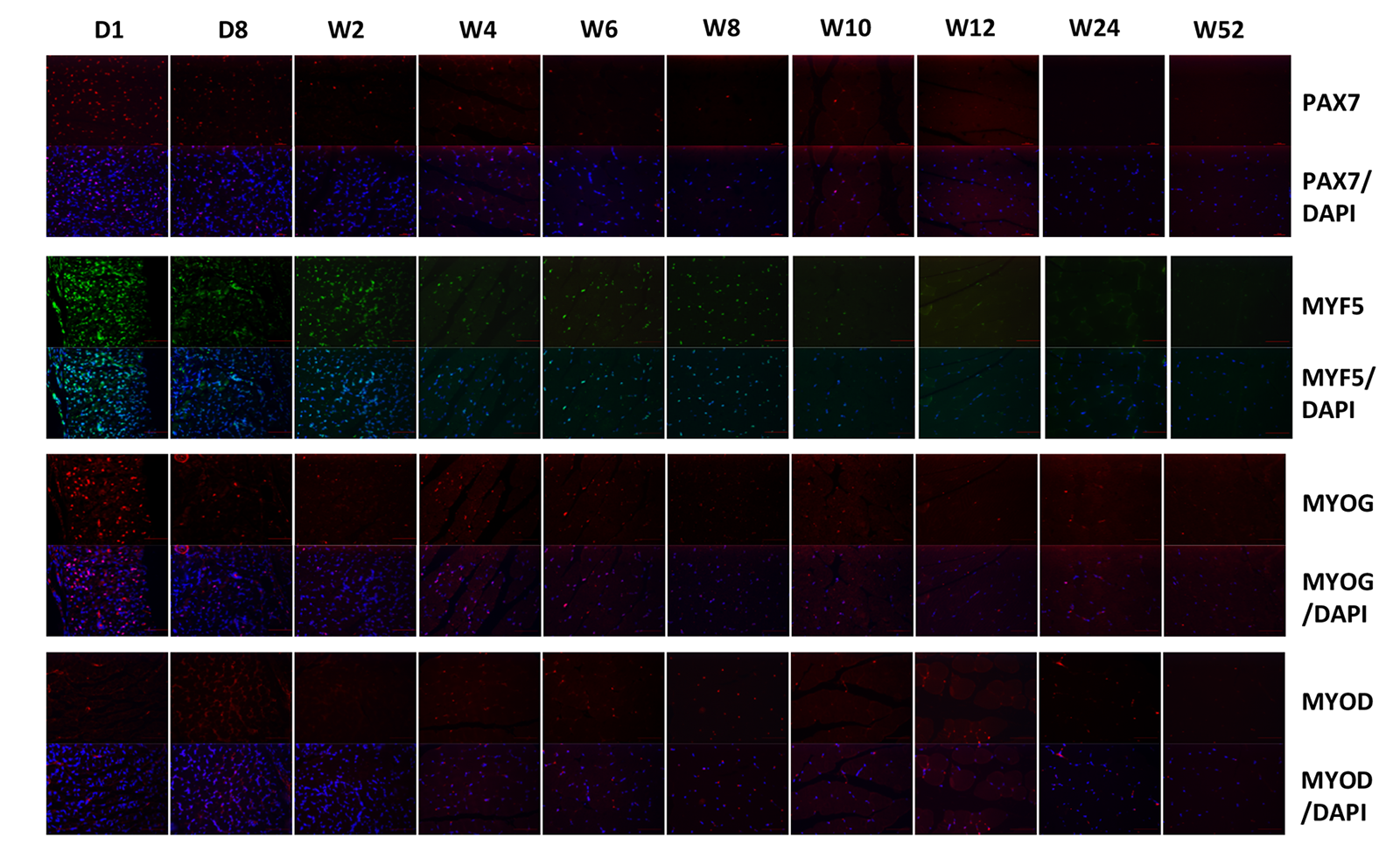

Supplement: Supplementary file 1 [file ACEL-17-na-s001.tif]

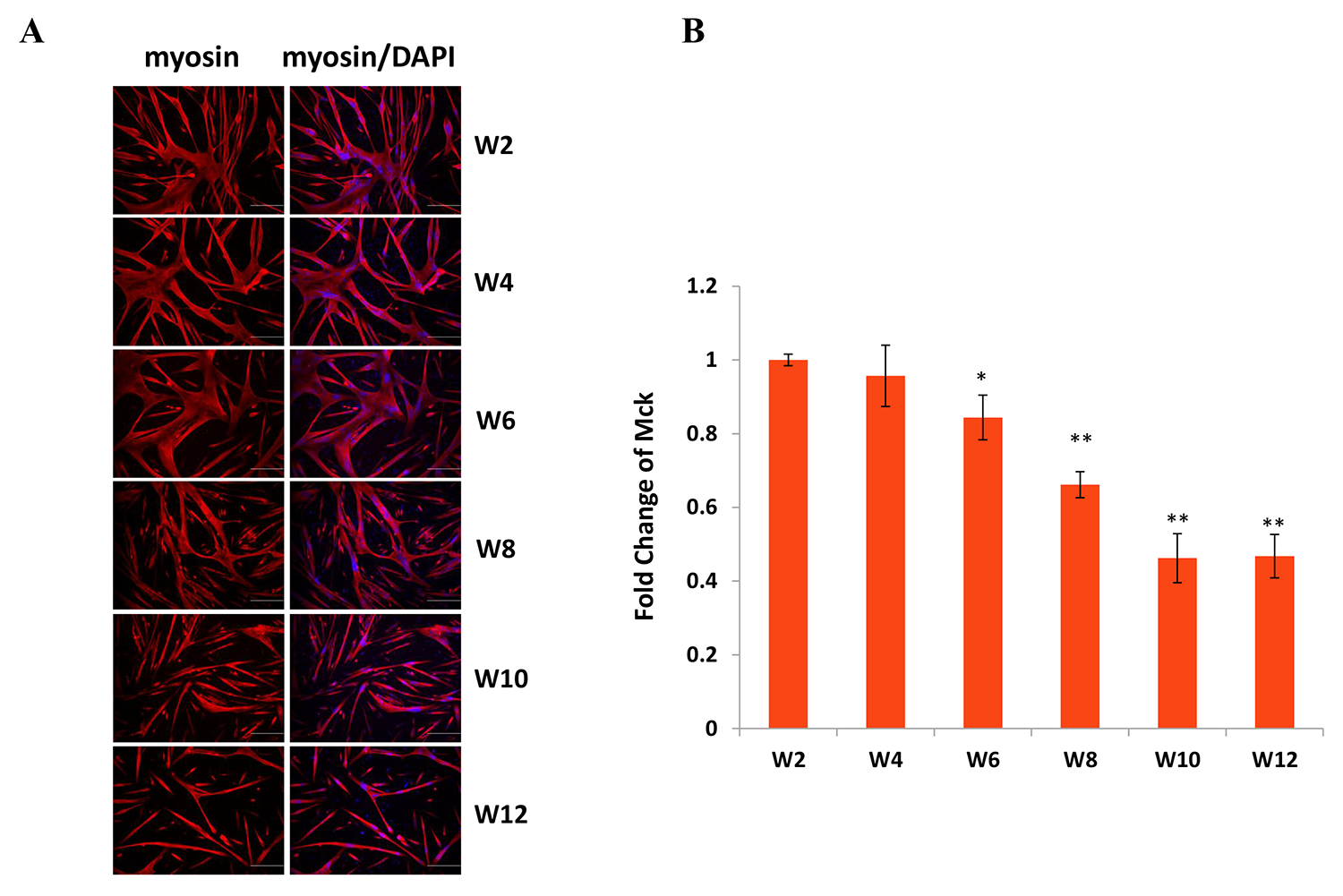

Supplement: Supplementary file 2 [file ACEL-17-na-s002.tif]

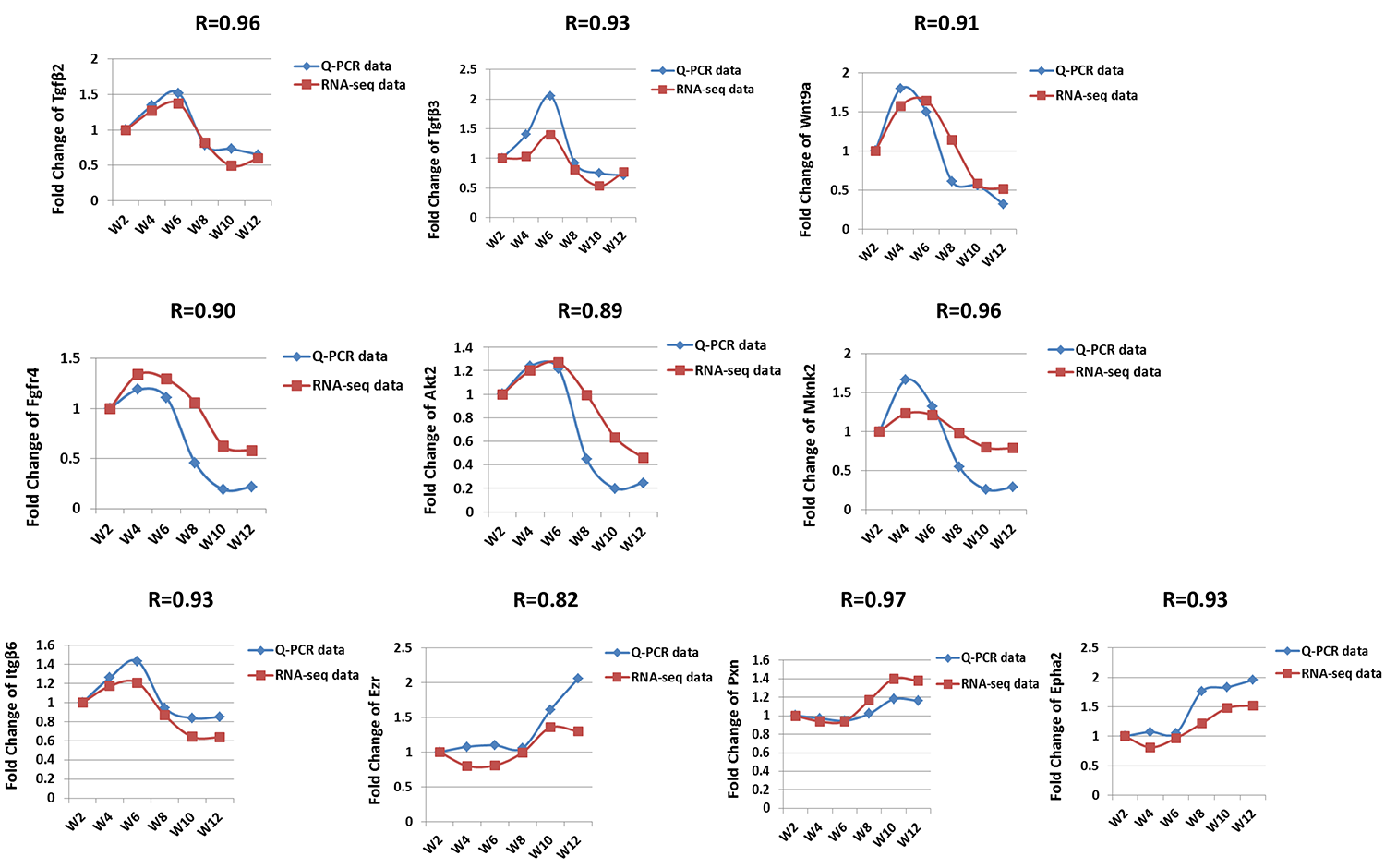

Supplement: Supplementary file 3 [file ACEL-17-na-s003.tif]

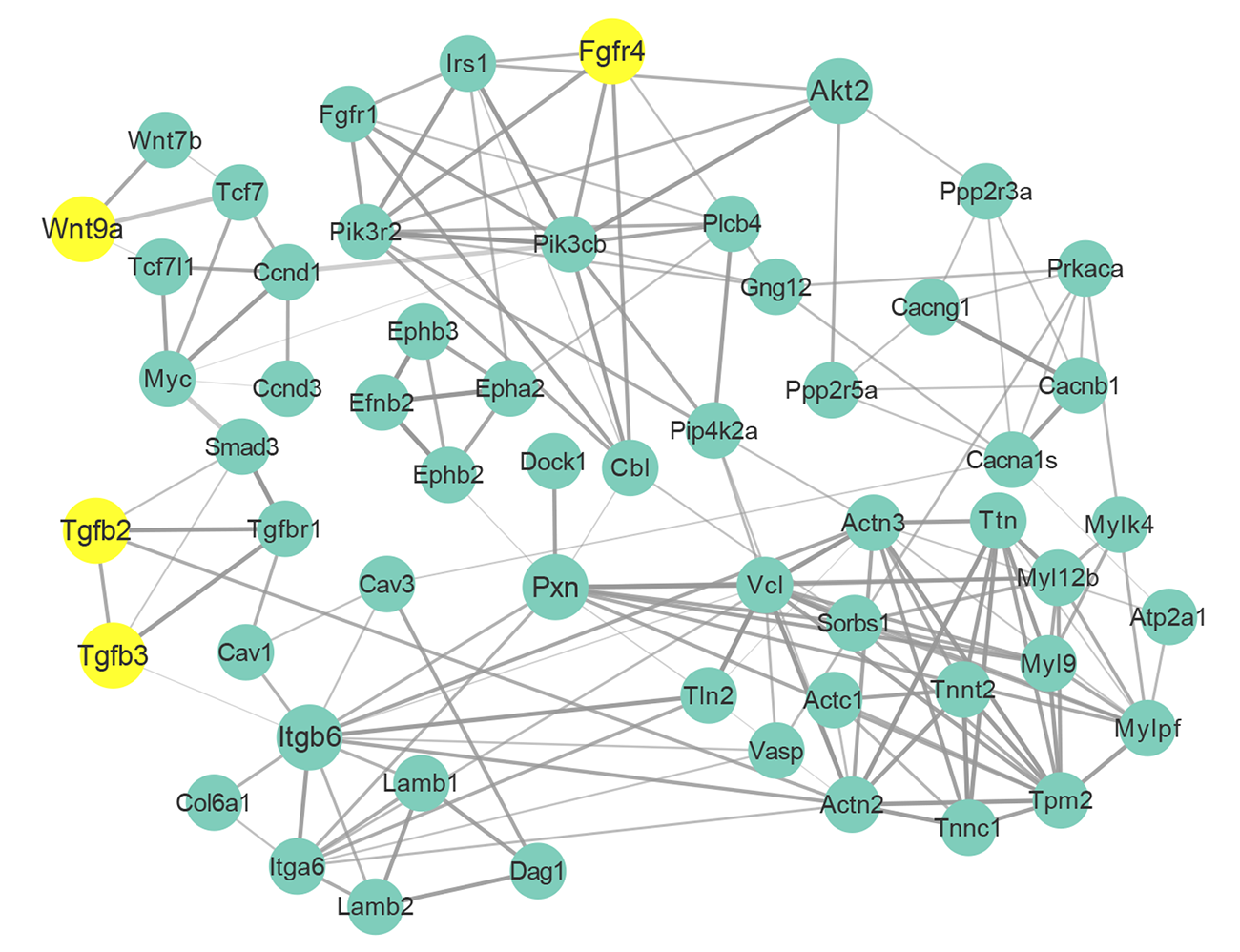

Supplement: Supplementary file 4 [file ACEL-17-na-s004.tif]

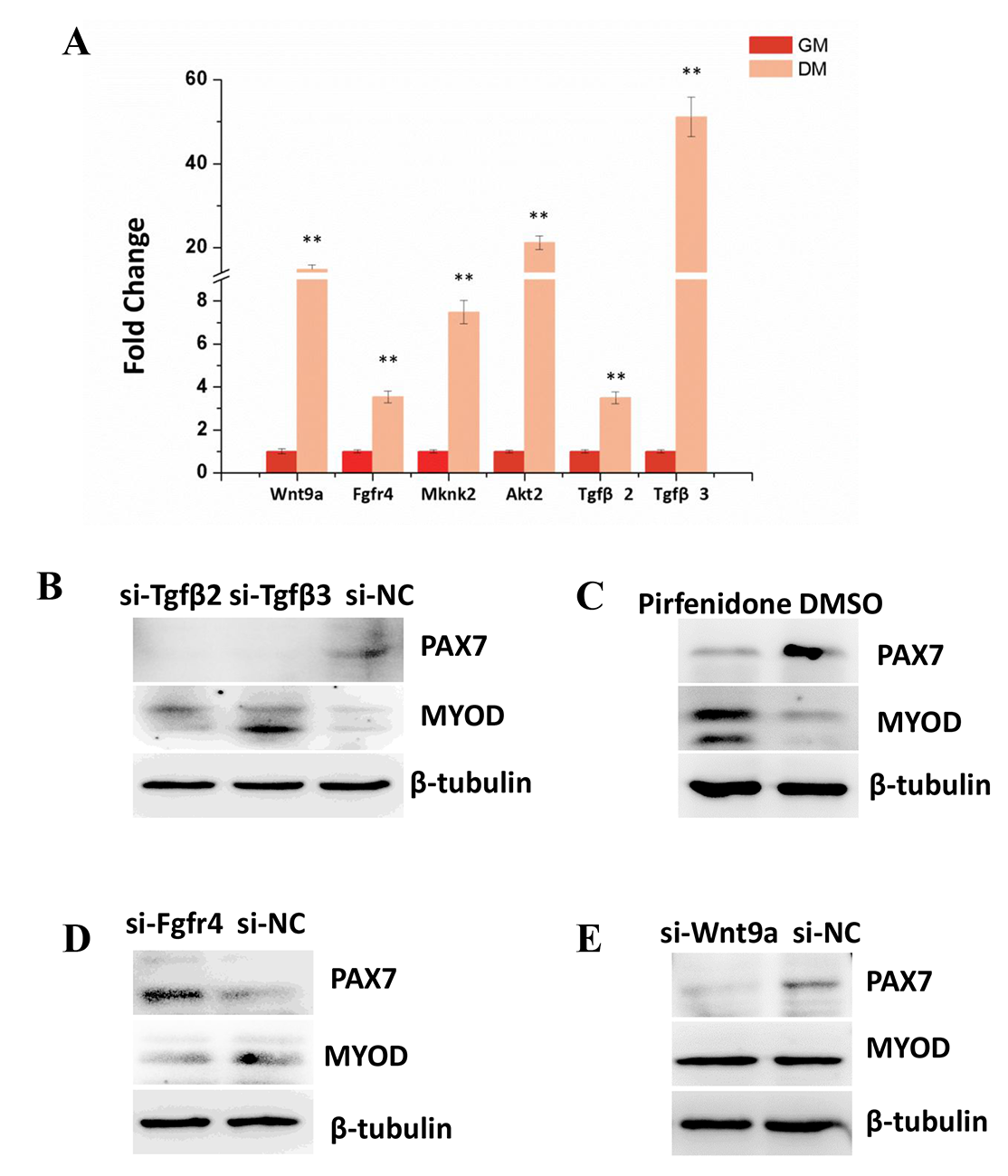

Supplement: Supplementary file 5 [file ACEL-17-na-s005.tif]
