# Supplementary material for: Synergistic effects of TGFβ2, WNT9a, and FGFR4 signals attenuate satellite cell differentiation during skeletal muscle development
Source: Aging Cell. 2018 Jun 4;17(4):e12788. doi: 10.1111/acel.12788 (PMC6052404; doi:10.1111/acel.12788)
Supplement: Supplementary file 6 [file ACEL-17-na-s006.docx]

**Supplementary Figure Legends**

**Figure S1.** Expression pattern of myogenic factors in skeletal muscle development. Paraffin section immunofluorescence was performed to test the expression pattern of PAX7, MYF5, Myogenin (MYOG), and MYOD in gastrocnemius muscle of mice at different time points (Day1, Day8, Week2, Week4, Week6, Week8, Week10, Week12, Week24, and Week52). Scale bars: 50 μm. Magnification: 400×.

**Figure S2.** (A) Satellite cells were induced differentiation for 48h, and immunofluorescence staining was performed to detect myosin (red). Cell nucleus was stained blue by DAPI. Scale bars: 200 μm. Magnification: 100×. (B) Q-PCR was performed to test the fold change of *Mck* expression in satellite cells after differentiation. *Tubulin* was used as the internal control, and the relative fold change was compared to the expression in Week 2 satellite cells. Triplicate samples were analyzed for each treatment, and the results were presented as the mean±s.e.m. *, P<0.05; **P<0.01.

**Figure S3.** The correlation index between RNA-seq data and Q-PCR data.

**Figure S4.** Analysis of the protein interacted between genes using String software according to protein interplay index (confidence>0.7). Genes were selected by the number of interaction gene (n>3).

**Figure S5.** (A) Q-PCR was performed to detect the difference expression of *Tgfβ2*, *Tgfβ3*, *Wnt9a*, *Fgfr4*, *Akt2*, and *Mknk2* genes in Week4 satellite cells between proliferation and differentiation status. *Tubulin* was used as the internal control, and the relative fold change was compared to the expression in proliferative cells. Triplicate samples were analyzed for each treatment, and the results were presented as the mean±s.e.m. *, P<0.05; **P<0.01. (B-C) Western blot results of PAX7 and MYOD in proliferative satellite cells when TGFβ2 and TGFβ3 was inhibited using siRNA (B) or pirfenidone (C). (D) Western blot results of PAX7 and MYOD in proliferative satellite cells when FGFR4 was inhibited using siRNA. (E) Western blot results of PAX7 and MYOD in proliferative satellite cells when WNT9a was inhibited using siRNA. *Tubulin* was used as the internal control.

**Table S1. Primary Antibodies for** [**immunofluorescence**](file:///D:\tools\youdao\7.1.0.0421\resultui\dict\?keyword=immunofluorescence) **and western blot**

| Primary Antibody | Antigen | Application | Working concentration | Manufacturer | Product code |
| --- | --- | --- | --- | --- | --- |
| Anti-Pax7 mouse | PAX7 | IF, WB | 1:100 dilution | DSHB, USA |  |
| Anti-Laminin rabbit | laminin | IF | 1:500 dilution | Sigma Life Science, USA | L9393 |
| Anti-Myf5 rabbit | MYF5 | IF, WB | 1:50 dilution | Santa Cruz, USA | sc-302 |
| Anti-Myogenin mouse | myogenin | IF | 1:100 dilution | Abcam, USA | ab1835 |
| Anti-MyoD (M-318) rabbit | MYOD | IF | 1:100 dilution | Santa Cruz, USA | sc-760 |
| Anti-myosin(skeletal, fast) mouse | myosin | IF | 1:200 dilution | Sigma Life Science, USA | M4276 |
| MNK2 antibody rabbit | MKNK2 | WB | 1:500 dilution | GeneTex, USA | GTX34078 |
| Phospho-AKT2-S474 Polyclonal Antibody rabbit | p-AKT2 | WB | 1:500 dilution | ABclonal, P.R.C | AP0305 |
| WNT9A Polyclonal Antibody rabbit | WNT9a | WB | 1:500 dilution | ABclonal, P.R.C | A7939 |
| TGFB2 Polyclonal Antibody rabbit | TGFβ2 | WB | 1:500 dilution | ABclonal, P.R.C | A3640 |
| TGFB3 Polyclonal Antibody rabbit | TGFβ3 | WB | 1:500 dilution | ABclonal, P.R.C | A8460 |
| FGFR4 antibody rabbit | FGFR4 | WB | 1:500 dilution | GeneTex, USA | GTX105022 |
| AKT2 Rabbit PolyAb | AKT2 | WB | 1:500 dilution | protein tech, USA | 17609-1-AP |
| β-tubulin mouse monoclonal antibody | β-tubulin | WB | 1:5000 dilution | Sungene Biotech, P.R.C | KM9003T |

**Table S2. Primers for Q-PCR in this study.**

| Name | Sequence (5' - 3') |
| --- | --- |
| MyHC2d-F | GGACCCACGGTCGAAGTTG |
| MyHC2d-R | CCCGAAAACGGCCATCT |
| Mck-F | GCTTATGGTGGAGATGGAGA |
| Mck-R | GGCCATCACGGACTTTTATT |
| β-tubulin-F | GACTATGGACTCCGTTCGCTC |
| β-tubulin-R | TATTCTTCCCGGATCTTGCTG |
| Tgfβ2-F | CTGTACCTTCGTGCCGTCTAATA |
| Tgfβ2-R | GACTGTTGTGACTCCAGTCTGTAGG |
| Tgfβ3-F | CAACATGGTGGTGAAGTCGTG |
| Tgfβ3-R | GAGGTCTGTCGCTTTGGTTTT |
| Wnt9a-F | GATTTGCGAGCCCGAGTG |
| Wnt9a-R | GTCTCATATTTGTGTTTTAGGTGCTT |
| Itgβ6-F | GGGGGTGTCACTGGCGAT |
| Itgβ6-R | GAGGATTGGTTCCCGTTTGC |
| Akt2-F | CCAGATGGTCGCCAACAGT |
| Akt2-R | CTCCTTGCGCAGGATCTTC |
| Fgfr4-F | CTGACTCGCAGACGACATGAG |
| Fgfr4-R | AGGCCATGATCTTGAGATGAGA |
| Ezr-F | CCAAGCAACGCATTGACGA |
| Ezr-R | TCCAAACAGGAAGTGATTCGGTA |
| Mknk2-F | GAATGAGACATGATCCTGGGCT |
| Mknk2-R | TTGGTGGGAAAGGAGTCCG |
| Pxn-F | TCCAGAAGGCTTCCACGAGA |
| Pxn-R | CTGCCCGTCGTGCTCAAA |
| Epha2-F | CTACTACAAGAAGTGTCCCGAGATG |
| Epha2-R | AGACTTGAAGAATCCTGGAGAACA |
